# Scala vestibuli cochlear implant supported by 3D modeling of the inner ear

**DOI:** 10.1007/s00508-021-01935-7

**Published:** 2021-09-03

**Authors:** Clemens Holzmeister, Alexandros Andrianakis, Peter Kiss, Ulrich Moser, Matthias Graupp

**Affiliations:** Department on Otorhinolaryngology, Head and Neck Surgery, Medical University of Graz, Auenbruggerplatz 26, 8036 Graz, Austria

**Keywords:** Hearing loss, Scala tympani, Sensorineural, Imaging, Rehabilitation

## Abstract

Patients with scala tympani (ST) ossification present a distinct surgical challenge. Three-dimensional (3D) segmentation of the inner ear offers accurate identification of ossification and surgical planning of the cochleostomy to access the scala vestibuli. The scala vestibuli placement of cochlear implantation electrode is an alternate solution in these patients and is well supported by the literature.

The present report describes a case of cochlear implantation in the scala vestibuli assisted by 3D segmentation of the cochlea for a patient with ossification in the ST and reviews the relevant literature. Clinical presentation of a 45-year-old Austrian female who was referred with a history of sudden sensorineural hearing loss 2 years ago in the right ear, confirmed by pure tone audiometry (PTA) and acoustically evoked auditory brainstem response (ABR). 3D segmentation of the inner ear identified the extent of ossification in the ST and assisted in the surgical planning of cochleostomy drilling anterior-superior to the round window to access the scala vestibuli for the electrode placement. Postoperative computed tomography (CT) to confirm the electrode placement in the scala vestibuli and PTA was performed to assess the hearing threshold following the cochlear implantation. Postoperative CT confirmed the full insertion of a flexible electrode. The hearing threshold measured by PTA was ≤ 40 dB across all frequencies tested. Review of the literature identified a total of 13 published reports on cochlear implantation electrode placement in scala vestibuli in cases with ossification in the ST.

## Introduction

Sudden sensorineural hearing loss (SSNHL) is an unexplained, rapid loss of hearing either all at once or over a few days and often affects only one ear [1]. During 2006 and 2007, the annual incidence of SSNHL was 27 per 100,000 patients in the USA and this incidence rate increases with increasing age, ranging from 11 per 100,000 in patients younger than 18 years to 77 per 100,000 in patients 65 years and older [2]. An SSNHL can be the result of variety of reasons including infections, head trauma, autoimmune diseases, exposure to certain drugs that treat cancer or severe infections, blood circulation problems and neurological disorders such as multiple sclerosis [3]. Labyrinthitis ossificans is often a side effect of some of the SSNHL causes and creates surgical challenges in the placement of cochlear implant (CI) electrode.

Labyrinthitis ossificans and how far it extends inside the cochlear lumen can be visualized by computed tomography (CT) images; however, it could be challenging for young and inexperienced surgeons and or radiologists to compile the entire series of CT scans in bringing a three-dimensional (3D) representation of the anatomical structure in mind. 3D segmentation of the inner ear has been applied clinically in cases with inner ear malformations to accurately identify anatomical structures available, thereby simplifying the CI electrode placement [4–8]. The same technique could be applied in cases with different degrees of scala tympani (ST) ossification to identify how deep it has extended and this helps surgeons to decide the placement of the CI electrode either in ST or in scala vestibuli (SV).

This article reports a case of ST ossification as identified by the 3D segmentation of the inner ear. We found the 3D segmentation of the inner ear structures clinically useful in making the decision of placing the electrode in SV and as well in the surgical planning of the cochleostomy to access the SV. The review of the relevant literature supported SV electrode placement in cases with difficult if not impossible ST electrode placement due to cochlear ossification.

## Clinical presentation

A 45-year-old Austrian female was referred to the ear, nose and throat (ENT) department of the Medical University of Graz, Austria for CI, after she had been diagnosed as deaf in the right ear2 years ago due to SSNHL. The left ear showed no indications of hearing loss. All investigations performed elsewhere at the time of SSNHL, including MRI, showed normal results.

### Intervention

The ENT examination at the time of consultation at our department including ear microscopy showed an inconspicuous finding. Audiological examination including acoustically evoked auditory brain stem evoked response (ABR) and pure tone audiometry confirmed deafness in the right ear. The preoperative CT image dataset was visualized using 3D slicer (https://www.slicer.org/; version 4.11.0, Boston, MA, USA), an advanced DICOM viewer that provides the possibility to 3D segment the anatomical structures of our interest. The 3D segmentation of the fluid-filled inner ear structures and the portion of ossification in the ST were done separately by setting different grey scale thresholds to capture the corresponding structures as accurately as possible. The steps involved in 3D segmentation of the inner ear structures are described elsewhere in detail [4]. The patient underwent CI placement at the ENT department of Medical University of Graz, Austria.

### Outcome measures

Postoperative CT was immediately performed to confirm the electrode placement inside the SV and pure tone audiometry (PTA) was performed to assess the hearing threshold 1 month following the CI.

### Literature review

**Fig. 1:**
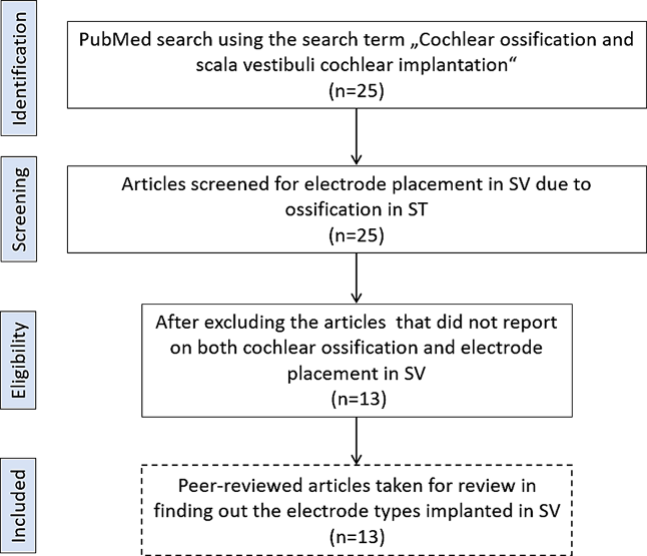
Flowchart describing the steps taken in the systematic review of literature in finding the electrode types placed in the SV in cases with ST ossification, from all those earlier reports

To perform the review of the literature on the relevant topic, a MEDLINE search was performed through the United States National Library of Medicine’s PubMed online database. Using the combined search terms cochlear ossification and scala vestibuli cochlear implantation with results limited to the English language, 25 articles were identified. Relevant case reports and series were examined for patients with ossification in ST and CI electrode placement in SV. Labyrinthitis ossification without CI, new bone formation after CI, delayed hearing preservation after CI, SV electrode placement without ossification in ST and electrode scalar deviation to SV were excluded from analysis. Eligible articles were reviewed to extract the electrode type implanted in the SV for comparison of the electrode type implanted in the current case report. Fig. 1 is a flowchart that describes the steps taken in the systematic review of literature in finding the electrode types placed in the SV in cases with ST ossification.

## Results

**Fig. 2:**
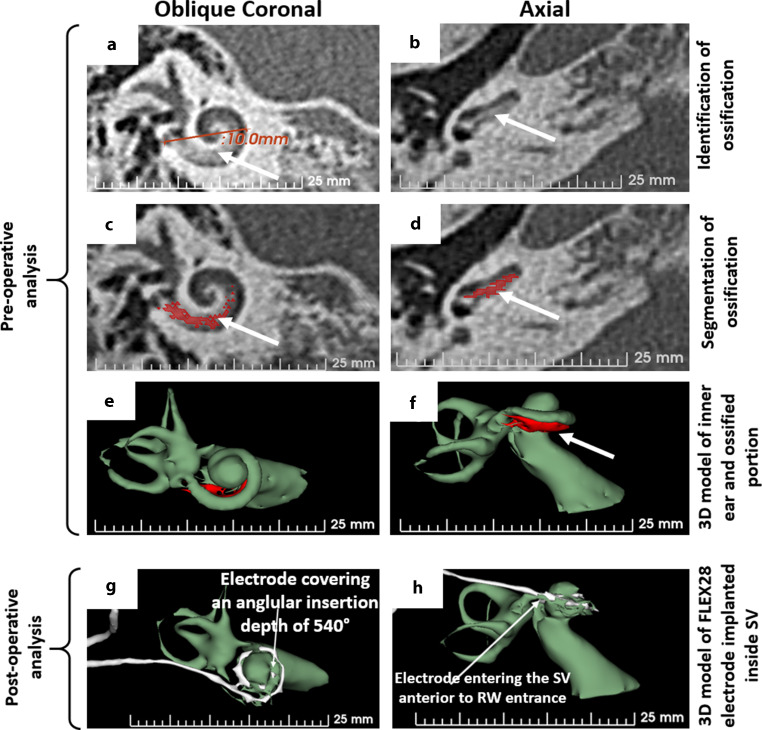
Visualization of the right ear in both oblique coronal and axial planes. Oblique coronal view of the cochlea with the basal turn diameter measured 10 mm and the white arrow pointing to basal ossification (**a**). Axial view showing the ossification in the basal portion of the ST (**b**). Segmentation of the ossified portion of ST (red shaded area) as seen in both coronal (**c**) and axial planes (**d**). 3D model (3D slicer) of the inner ear and ossification (red portion) as seen in both coronal (**e**) and axial planes (**f**). Analysis of the postoperative CT scans showed the 28 mm electrode array inserted to an AID of 540° (**g**) and the electrode fully placed in the SV (**h**)

The CT image analysis of the right temporal bone applying 3D segmentation of the inner ear structures showed ossification in the basal portion of the cochlea as shown in Fig. 2 (preoperative analysis). The extent of ossification was seen only in the ST and it was estimated to be around 9 mm from the RW entrance (Fig. 2a–d). The cochlear size as measured by the diameter of the basal turn in the oblique coronal plane was 10 mm as shown in Fig. 2a. In contrast to the ossification of ST, the SV was completely patent as seen in both 2D images and the 3D model (Fig. 2b, d and f). The 3D model of the inner ear (green structure) showing the ossified portion of the ST (red structure) was useful in planning the location of cochleostomy anterior superior to the RW to reach the SV. Following all the regular CI surgical steps to reach the RW, it was found to be ossified as confirmed by gently touching it with a pointed surgical tool. Cochleostomy drilling was then performed approximately 1.5 mm anterior and superior to the RW niche. After identifying the SV lumen, a dummy insertion electrode provided by the company MED-EL (Innsbruck, Austria) was inserted to confirm the extent of SV lumen for a length of 30 mm and was followed by the insertion of a functional electrode array of length 28 mm to its full prescribed length inside the SV. Postoperatively, the patient felt well, without any dizziness or vertigo and postoperative CT images confirmed correct placement of the electrode covering an AID of 540° inside the SV (Fig. 2g, h).

**Fig. 3:**
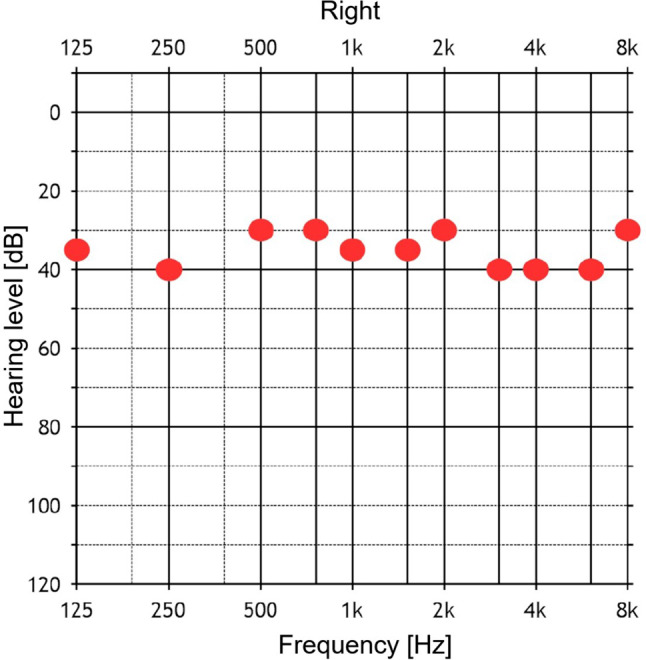
Postoperative pure tone audiogram of the implanted ear showing hearing threshold of ≤ 40 dB across all frequencies tested

The externally worn audio processor was activated 4 weeks following the CI surgery and the pure tone audiogram showed the hearing threshold ≤ 40 dB across all frequencies tested as shown in Fig. 3.

A review of the relevant literature from PubMed search identified 13 articles dated from 1990 until 2018 reporting on the SV placement of CI electrode array due to ossification in ST ([9–21]; Table 1). The etiology of hearing loss reported in these 13 articles ranged from meningitis, temporal bone fracture, otosclerosis, sudden SNHL, autoimmune disease, Noonan’s syndrome, Cogan syndrome, Meniere’s disease and inner ear malformations. The electrode array types implanted in all these cases mainly belonged to the older generation types that were intended to cover the basal turn (360° of angular depth) of the cochlea. The one case from Kiefer et al. reported on a full insertion of a STANDARD electrode from MED-EL that offers an insertion depth of 30 mm [11]. Although not given in Table 1, cases reported in all these studies showed an improved hearing performance after CI procedure with electrode in SV, compared to the preoperative hearing scores.

**Table 1:** Summary of data from the studies that reported on SV electrode placement

Study	Etiology of hearing loss	Number of cases reported with electrode in SV	Electrode type	Electrode insertion depth reported
Steenerson et al. (1990) [9]	Pneumococcal meningitis	2	Nucleus 22 channel	23 mm
Bird et al. (1999) [10]	Pneumococcal meningitis and previous implantation scar tissue	2	Clarion 1.2 device	n/a
Kiefer et al. (2001) [11]	Temporal bone fracture, severe otosclerosis, Noonan’s syndrome, inner ear malformation	4	Nucleus 22 channel, Nucleus 24M, MED-EL Short and STANDARD	12–30 mm
Ruckenstein et al. (2001) [12]	Otosclerosis	2	Nucleus 24M and Clarion	n/a
Pasanisi et al. (2002) [13]	Ossification	11	Nucleus 24M	n/a
Bacciu et al. (2002) [14]	Hereditary, meningitis, otosclerosis, autoimmune	10	Nucleus 22 and Nucleus 24M	n/a
Berrettini et al. (2002) [15]	Cogan syndrome	2	Nucleus 24M	≈17 mm
Pasanisi et al. (2003) [16]	Cogan syndrome	2	Nucleus Contour and Nucleus 24M	n/a
Reeck et al. (2003) [17]	Meningitis, Idiopathic thrombocytopenic purpura	1	Clarion	–
Lin et al. (2008) [18]	Meningitis and unknown	11	MED-EL STANDARD	31 mm
Nichani et al. (2011) [19]	Bacterial meningitis	3	MED-EL COMPRESSED	n/a
Vashishth et al. (2017) [20]	Meningitis	1	n/a	n/a
Trudel et al. (2018) [21]	Otosclerosis, sudden SNHL, auditory neuropathy	21	Contour Advance, Slim Straight, HiFocus 1J, Mid-Scala	Covers mainly the basal turn of the cochlea

n/a data not given

## Discussion

The SV electrode placement was first reported by Steenerson et al. in 1990 as an alternative solution, when the ST is occluded with ossification [9]. Since then there have been 13 reports up to 2018, that have demonstrated the SV electrode placement whenever ST was not available due to ossification [9–21]. Kiefer et al. [11] and Lin et al. [18] were the only reports to show deep insertion of an older generation electrode in the SV. Other than these two reports, all the other reports given in Table 1, reported on the short electrode array placement in the SV that literally covered not more than 360° of AID. To the best of the authors’ knowledge, this is the first report showing the full insertion of the flexible version of a longer length electrode array (28 mm) covering an AID of 540° in SV due to ossification in the ST.

**Fig. 4:**
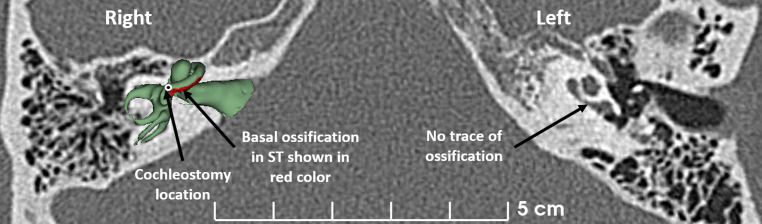
Three dimensional segmented inner ear image of the right ear overlaid on the CT image slice (3D slicer). It shows the basal ossification in red color and the cochleostomy location as pointed by the white circle around a black dot. The left cochlea shows no traces of ossification

The 3D segmentation of the inner ear (green) and the ossified portion (red) that were overlaid on the 2D image slice gave us a clear picture of where to drill the cochleostomy to access the SV. Fig. 4 showcases the 3D segmented image of the right ear along with the ossified portion in the basal turn of the ST. The white circle around the black dot in Fig. 4 pointed by the black arrow is the location identified for cochleostomy drilling to access the SV. The left cochlea did not show any traces of ossification anywhere within the cochlea.

Although there were earlier studies that reported on the clinical application of 3D segmentation of the inner ear especially in the identification of inner ear malformation types [4–8], to the best of our knowledge ours is the first report that applied 3D segmentation of the inner ear from the CT images, in accurately identifying the ossified portion of ST as shown in Figs. 2 and 4. The time required to perform the 3D segmentation is not more than 10–15 min and it was very helpful to us as operating surgeons in accurately accessing the SV by drilling the cochleostomy 1.5 mm anterior superior to the RW niche. The insertion of the dummy insertion electrode to a length of 30 mm inside the SV convinced us to choose a 28 mm long electrode and flexible array. Also, for the reason that this patient was profoundly deaf across all frequencies tested preoperatively, electrical stimulation across the entire frequency range was thought to be necessary and as supported by recent reports [22–24].

The postoperative image and the 3D segmentation of the implanted electrode confirmed the full insertion of the 28 mm long electrode array in SV without deviating back into the ST at any location along the cochlea lumen. The postoperative pure tone audiometry showing the hearing thresholds ≤ 40 dB across all frequencies tested confirmed the electrical stimulation of the neuronal elements from the SV.

The electrode placement in the SV due to ST ossification described in this report is in accordance with the literature findings. We felt that the 3D segmentation of the inner ear was clinically useful in understanding the extent of ST ossification, identification of the cochleostomy location to access SV for the electrode placement and as well for teaching our resident doctors. One of the limitations associated with this report is that no detailed information on the postoperative speech performance of the case presented was reported; however, subjectively the patient was highly satisfied with her overall hearing with CI in the right ear and natural hearing in the left ear. The literature from the past has taught us that the required levels of electrical stimulation for subjects implanted via the SV were similar to those required for subjects who had the standard ST insertion of the electrode array [19, 21]. Also, the literature has demonstrated that SV insertion of CI electrode offers hearing function comparable to ST insertion and SV is often available when the ST is not [14, 19, 21]. Although our case is only few weeks with the audio processor activated, the hearing threshold of ≤ 40 dB as measured from the pure tone audiometry is a clue that the patient will benefit from the CI procedure.

## Conclusion

This report demonstrates the usefulness of 3D segmentation of the inner ear in accurately identifying the ossified portion of the ST and the cochleostomy site to access SV. Insertion of the dummy insertion electrode to check the extent of SV availability was useful in deciding the length of the flexible electrode array. This report could add to the growing literature supporting SV electrode placement in cases with ossification in ST that cannot be drilled out completely.

## References

[1] Chandrasekhar SS, Tsai Do BS, Schwartz SR (2019). Clinical practice guideline: sudden hearing loss (update). Otolaryngol Head Neck Surg.

[2] Alexander TH, Harris JP (2013). Incidence of sudden sensorineural hearing loss. Otol Neurotol.

[3] Young YH (2020). Contemporary review of the causes and differential diagnosis of sudden sensorineural hearing loss. Int J Audiol.

[4] Dhanasingh A, Dietz A, Jolly C, Roland P (2019). Human inner-ear malformation types captured in 3D. J Int Adv Otol.

[5] Dhanasingh A (2019). Variations in the size and shape of human cochlear malformation types. Anat Rec (Hoboken).

[6] Alenzi S, Dhanasingh A, Alanazi H, Alsanosi A, Hagr A (2020). Diagnostic value of 3D segmentation in understanding the anatomy of human inner ear including malformation types. Ear Nose Throat J.

[7] Weiss NM, Langner S, Mlynski R, Roland P, Dhanasingh A (2021). Evaluating common cavity cochlear deformities using CT images and 3D reconstruction. Laryngoscope.

[8] Halawani RT, Dhanasingh A (2020). New classification of cochlear hypoplasia type malformation: relevance in cochlear implantation. J Int Adv Otol.

[9] Steenerson RL, Gary LB, Wynens MS (1990). Scala vestibuli cochlear implantation for labyrinthine ossification. Am J Otol.

[10] Bird PA, Balkany TJ, Hodges AV, Butts S, Gomez O, Lee D (1999). Using the CLARION cochlear implant in cochlear ossification. Ann Otol Rhinol Laryngol Suppl.

[11] Kiefer J, Weber A, Pfennigdorff T, von Ilberg C (2000). Scala vestibuli insertion in cochlear implantation: a valuable alternative for cases with obstructed scala tympani. ORL J Otorhinolaryngol Relat Spec.

[12] Ruckenstein MJ, Rafter KO, Montes M, Bigelow DC (2001). Management of far advanced otosclerosis in the era of cochlear implantation. Otol Neurotol.

[13] Pasanisi E, Bacciu A, Vincenti V (2002). Multi-channel cochlear implant in cochlear ossification. Acta Otorhinolaryngol Ital.

[14] Bacciu S, Bacciu A, Pasanisi E (2002). Nucleus multichannel cochlear implantation in partially ossified cochleas using the Steenerson procedure. Otol Neurotol.

[15] Berrettini S, Forli F, Neri E, Segnini G, Franceschini SS (2002). Scala vestibuli cochlear implantation in patients with partially ossified cochleas. J Laryngol Otol.

[16] Pasanisi E, Vincenti V, Bacciu A (2003). Cochlear implantation and Cogan syndrome. Otol Neurotol.

[17] Reeck JB, Lalwani AK (2003). Isolated vestibular ossification after meningitis associated with sensorineural hearing loss. Otol Neurotol.

[18] Lin YS (2009). Clinical outcomes of scala vestibuli cochlear implantation in children with partial labyrinthine ossification. Acta Otolaryngol.

[19] Nichani J, Green K, Hans P, Bruce I, Henderson L, Ramsden R (2011). Cochlear implantation after bacterial meningitis in children: outcomes in ossified and nonossified cochleas. Otol Neurotol.

[20] Vashishth A, Fulcheri A, Rossi G, Prasad SC, Caruso A, Sanna M (2017). Cochlear implantation in otosclerosis: surgical and auditory outcomes with a brief on facial nerve stimulation. Otol Neurotol.

[21] Trudel M, Côté M, Philippon D, Simonyan D, Villemure-Poliquin N, Bussières R (2018). Comparative impacts of scala vestibuli versus scala tympani cochlear implantation on auditory performances and programming parameters in partially ossified cochleae. Otol Neurotol.

[22] Canfarotta MW, Dillon MT, Buchman CA (2020). Long-term influence of electrode array length on speech recognition in cochlear implant users. Laryngoscope.

[23] Büchner A, Illg A, Majdani O, Lenarz T (2017). Investigation of the effect of cochlear implant electrode length on speech comprehension in quiet and noise compared with the results with users of electro-acoustic-stimulation, a retrospective analysis. PLoS One.

[24] O’Connell BP, Hunter JB, Gifford RH (2016). Electrode location and audiologic performance after cochlear implantation: a comparative study between nucleus CI422 and CI512 electrode arrays. Otol Neurotol.

